# Transgelin Contributes to a Poor Response of Metastatic Renal Cell Carcinoma to Sunitinib Treatment

**DOI:** 10.3390/biomedicines9091145

**Published:** 2021-09-03

**Authors:** Pavla Bouchalova, Jindrich Beranek, Petr Lapcik, David Potesil, Jan Podhorec, Alexandr Poprach, Pavel Bouchal

**Affiliations:** 1 Department of Biochemistry, Faculty of Science, Masaryk University, 625 00 Brno, Czech Republic; bouchalova@mail.muni.cz (P.B.); 451817@mail.muni.cz (J.B.); 409180@mail.muni.cz (P.L.); 2 Proteomics Core Facility, Central European Institute of Technology, Masaryk University, 625 00 Brno, Czech Republic; dpotesil@mail.muni.cz; 3 Department of Comprehensive Cancer Care, Masaryk Memorial Cancer Institute, 656 53 Brno, Czech Republic; jan.podhorec@mou.cz (J.P.); poprach@mou.cz (A.P.); 4 Department of Comprehensive Cancer Care, Faculty of Medicine, Masaryk University, 656 53 Brno, Czech Republic

**Keywords:** mccRCC, sunitinib, resistance, DIA-MS, transgelin

## Abstract

Renal cell carcinoma (RCC) represents about 2–3% of all cancers with over 400,000 new cases per year. Sunitinib, a vascular endothelial growth factor tyrosine kinase receptor inhibitor, has been used mainly for first-line treatment of metastatic clear-cell RCC with good or intermediate prognosis. However, about one-third of metastatic RCC patients do not respond to sunitinib, leading to disease progression. Here, we aim to find and characterize proteins associated with poor sunitinib response in a pilot proteomics study. Sixteen RCC tumors from patients responding (8) vs. non-responding (8) to sunitinib 3 months after treatment initiation were analyzed using data-independent acquisition mass spectrometry, together with their adjacent non-cancerous tissues. Proteomics analysis quantified 1996 protein groups (FDR = 0.01) and revealed 27 proteins deregulated between tumors non-responding vs. responding to sunitinib, representing a pattern of deregulated proteins potentially contributing to sunitinib resistance. Gene set enrichment analysis showed an up-regulation of epithelial-to-mesenchymal transition with transgelin as one of the most significantly abundant proteins. Transgelin expression was silenced by CRISPR/Cas9 and RNA interference, and the cells with reduced transgelin level exhibited significantly slower proliferation. Our data indicate that transgelin is an essential protein supporting RCC cell proliferation, which could contribute to intrinsic sunitinib resistance.

## 1. Introduction

Renal cell carcinoma (RCC) is a serious oncological disease newly affecting over 400,000 people per year worldwide with the highest incidence in Europe and North America, and substantially higher (two-thirds) prevalence in men in comparison to women. The incidence of RCC is increasing through the years, and the disease leads to more than 175,000 deaths per year [1]. From the histological point of view, RCC is rather a collection of different types of tumors, each derived from the various parts of the nephron and possessing distinct genetic characteristic, histological features and, to some extent, clinical phenotypes [2]. The most prevalent RCC type is clear-cell RCC (ccRCC) revealed in approximately 75–80% of all RCC. The management of RCC is mainly based on complete or partial nephrectomy with a subsequent targeted drug treatment [2] as chemotherapy and radiotherapy are not successful [3]. The RCC tumors are highly vascularized due to a high expression of hypoxia-induced factor (HIF1) further supported by von Hippel-Lindau gene (VHL) inactivation [4,5]. If ccRCC generalizes to metastatic ccRCC (mccRCC), targeted therapy goes on the scene. Vascular endothelial growth factor (VEGF) tyrosine kinase inhibitors (TKI) have been drugs of the first choice for years, namely in the first-line treatment of patients with good or intermediate risk according to Memorial Sloane Kettering Cancer Centre (MSKCC) score. Anti-programmed death (PD1)/programmed death-ligand 1 (PD-L1) immunotherapeutic monoclonal antibodies and mammalian target of rapamycin (mTOR) inhibitors are being used for mccRCC treatment as well. These three therapeutic modes complement each other in mRCC management, especially if the patient does not respond to the selected treatment mode [6]. 

The VEGF inhibitor sunitinib is a small-molecule, multi-targeting rTKI, which was FDA-approved for RCC treatment in 2006. Sunitinib has been identified as a potent inhibitor of VEGF receptors (VEGFR) 1, 2 and 3, receptor-type tyrosine-protein kinase FLT3, mast/stem-cell growth factor receptor KIT, platelet-derived growth factor receptors (PDGFR) α and β [7,8], as well as of colony-stimulating factor type 1 receptor and the glial cell-line-derived neurotrophic factor receptor (RET) [9]. Sunitinib showed a better therapeutic response (a longer progression-free survival and disease stabilization) in comparison to previously used interferon alpha treatment and became a first-line standard in mccRCC care [2,10,11]. In RCC, sunitinib blocks simultaneously VEGFR and PDGFR, reducing tumor vascularization and triggering tumor cell apoptosis, leading to tumor shrinkage [10]. As the sunitinib action is non-specific, many undesirable side effects as well as drug resistance have been observed. 

While 70% of tumors show initial response and may develop extrinsic (secondary) sunitinib resistance in the majority of the patients within 6–15 months of the treatment, 30% of RCC tumors demonstrate intrinsic sunitinib resistance [12]. The resistance to sunitinib emerges through different mechanisms: the intrinsic resistance seems to be related to the primary redundancy of available angiogenic signals from the tumor, causing unresponsiveness to VEGF-targeted therapies [13]. Hypoxia could contribute to intrinsic resistance by selecting a more malignant RCC phenotype, which may accelerate metastatic development and may make the cells prone to insensitivity for anti-angiogenic treatment [14,15]. The tumor cells can also overcome the noxious rTKI hypoxic microenvironment by switching to invasive epithelial-to-mesenchymal transition (EMT) phenotype [16]. The extrinsic resistance is caused by the activation of an angiogenic switch, leading either to up-regulation of the VEGF pathway, the recruitment of alternative factors responsible for tumor revascularization [17], or alternatively, to the sequestration of sunitinib in lysosomes, which reduces its bioavailability [18]. In addition, the sunitinib resistance may be further supported by EMT [19] and by elevated expression of interleukin-8 [20], or by extracellular matrix metalloproteinase inducer (EMMPRIN) [21], which are both induced with sunitinib treatment, supporting tumor growth and angiogenesis activity. The extrinsic sunitinib resistance is reversible [22], and the restoration of sensitivity to sunitinib and significantly longer progression-free survival are observed in patients who had a sunitinib-free interval longer than 6 months in comparison to those with a sunitinib-free interval shorter than 6 months [23].

**Figure 1:**
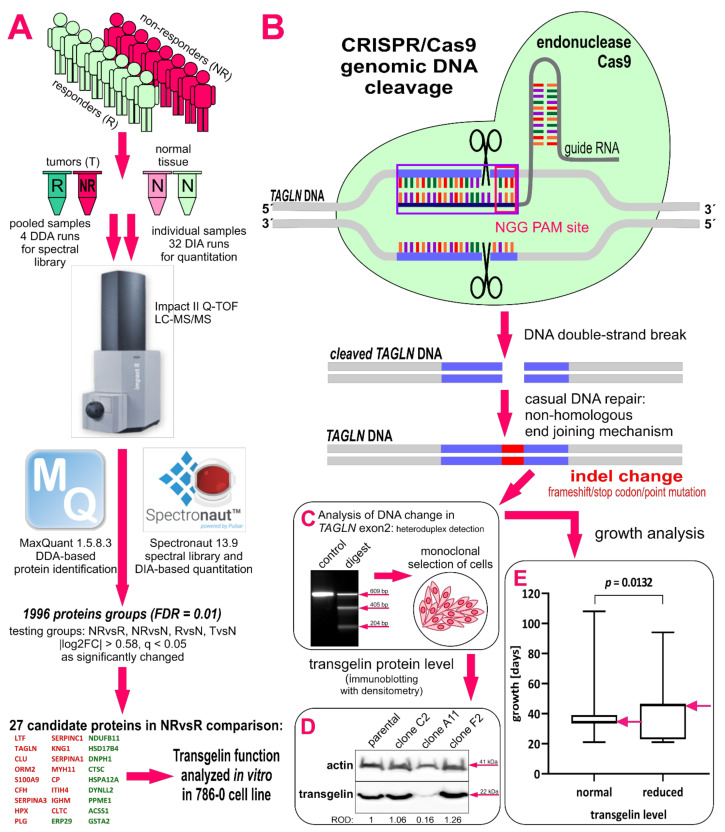
Workflow for the identification of potential biomarker targets and validation experiments. (**A**) Sixteen selected mccRCC tumors of patients responding (R) and non-responding (NR) to sunitinib with their adjacent normal renal tissue (N) were analyzed in 32 data-independent acquisition–mass spectrometry (DIA-MS) runs and as pooled samples in four data-dependent acquisitions (DDA) runs. Analysis of both DDA/DIA data revealed the presence and quantity of 1996 protein groups (FDR = 0.01). Proteins with |log2FC| > 0.58, q < 0.05 were considered statistically significant in comparisons between groups. A total of 27 proteins were identified as associated with sunitinib non-responsiveness; red genes were up-regulated, green genes were down-regulated. Transgelin (TAGLN) was selected for functional characterization based on quantitative results and its known characteristics. (**B**) CRISPR/Cas9 schematic overview: 786-0 cells were transfected with a complex of Cas9 and gRNA. gRNA targets the site in the gene exon and points Cas9 to cleave DNA upstream to protospacer adjacent motif (PAM), which is a necessary 3 nt long DNA sequence for Cas9 to cut. Two different gRNAs targeting two exon2 sites were used for *TAGLN* gene silencing. Double-strand breaks emerge in DNA and a non-homologous end-joining repairing mechanism inserts “indel” changes into *TAGLN* gene. This was confirmed by the detection of heteroduplexes (**C**), which only emerged during the hybridization of PCR products after change in DNA sequence (mix of parental and modified sequences). Then, a monoclonal selection of cells was performed in the cell population with changed DNA, and each viable clone was tested for transgelin protein level using immunoblotting (**D**). The transgelin/actin relative optical density (ROD) ratio was normalized to those of parental 786-0 cells. (**E**) Clones with reduced transgelin level (*n* = 27; ROD < 0.5) showed slower proliferation in comparison to clones with normal transgelin level (*n* = 96; ROD ≥ 0.5), the difference between medians (7.313 ± 2.908 days) was statistically significant. The pink arrows point to the medians of the groups. Representative gels are shown.

The aim of the presented pilot proteomics study is to identify potential protein biomarker(s) associated with intrinsic sunitinib resistance in mccRCC tumors that could potentially identify patients who would benefit from other treatment than sunitinib. This is of a great clinical importance due to the strong health side effects of sunitinib treatment. To achieve this goal, we performed a retrospective pilot proteomics study on 16 mccRCC tumor tissues and their non-cancerous counterparts, of which half responded to sunitinib three months after the treatment initiation and half did not (see Figure 1 for study workflow). We generated a RCC-specific spectral library and used data-independent acquisition mass spectrometry (DIA-MS) for the analysis of tissue profiles, as it provides a consistent quantification of tumor-derived proteins and peptides [24,25]. The function of the key protein, transgelin, was studied using CRISPR/Cas9 and RNA interference (RNAi) in cell lines derived from ccRCC tumors to confirm its role in RCC tumor development and sunitinib resistance.

## 2. Materials and Methods

### 2.1. Patients

Tissues from 16 patients with mccRCC who were treated at Masaryk Memorial Cancer Institute (MMCI) in Brno, Czech Republic in 2006–2018 with at least 5-years follow-up were involved in the study. All specimens were received within 20 min of surgical removal according to standardized MMCI protocol and immediately evaluated by a pathologist and snap frozen in liquid nitrogen. There were 10 men and 6 women in the study with a mean age of 63.7 ± 9.5 years. All patients underwent sunitinib treatment in the first line, and their therapy outcome was evaluated three months after the therapy initiation. Eight patients responded to sunitinib therapy (R; reached partial or complete remission) while another eight patients did not respond (NR; disease progressed). Both tumor and adjacent normal (N) tissues were available from each patient. The detailed clinicopathological characteristics of the patients are in Table 1 and Supplementary File S1. The study was approved by the ethics committee of MMCI (2018/150/MOU) and all patients have signed the informed consent.

### 2.2. Proteomics 

#### 2.2.1. Lysis of Tissue Samples

Approximately 2 × 2 × 2 mm pieces of kidney tissue were lysed in 200 µL of lysis buffer (6 M guanidine hydrochloride, 1% Triton X-100 in 0.1 M phosphate buffer pH 6.6) and mechanically homogenized 2 × 2 min at 25 s^−1^ in the homogenizer (Retsch, Haan, Germany). The homogenates were subsequently sonicated 30 × 0.1 s under 50 W power using needle sonication (Bandelin HD 2200; Bandelin, Berlin, Germany). The samples were left for 15 min at room temperature and then were centrifuged at 14,000 rpm/20 min/4 °C. The supernatants were transferred into a new tube, and RC-DC protein assay (Bio-Rad, Hercules, CA, USA) was used to measure protein concentrations in lysates.

#### 2.2.2. Preparing Samples for Mass Spectrometry (MS) Analysis

Trypsin digestion was performed using filter-aided sample preparation (FASP) [26] with several modifications: 100 µg of protein was added onto Vivacon 500 ultrafiltration spin columns (30 kDa membrane cutoff, Sartorius Stedim Biotech, Göttingen, Germany) with 200 µL of UA buffer (8 M urea in 0.1 M Tris/HCl, pH 8.5). The columns were centrifuged at 14,000× *g*/30 min/20 °C. Then, 100 μL of UA buffer was added onto the columns followed by the addition of 20 µL 0.1 M *tris*-(2-carboxyethyl)-phosphine, which was mixed and left in thermomixer (Eppendorf, Hamburg, Germany) for 30 min/600 rpm/37 °C to reach a complete reduction of proteins. Then, the samples were centrifuged at 14,000× *g*/15 min/20 °C. Subsequently, 100 μL of UA buffer and 20 μL of 0.3 M iodoacetamide were added onto the columns and mixed. The samples were alkylated for 1 min/600 rpm/25 °C in a thermomixer and then left for 20 min in the dark without shaking, which was followed by centrifugation at 14,000× *g*/15 min/20 °C. The columns were washed two times with 100 μL of 50 mM ammonium bicarbonate and centrifuged for 14,000× *g*/20 min/20 °C. The digestion was performed by the addition of 3.33 μL of 1 μg/μL trypsin (Promega, Madison, WI, USA) in 100 µL of 50 mM ammonium bicarbonate followed by incubation for 12 h at 37 °C in a wet chamber. The digests were collected by centrifugation at 14,000× *g*/15 min/20 °C. 

#### 2.2.3. Peptide Desalting Prior to LC-MS/MS

C18 Silica MicroSpin columns (NestGroup Inc., Southborough, MA, USA) were used to desalt the peptides prior to MS analysis. The columns were washed twice with 200 µL of 0.1% trifluoracetic acid (TFA) in acetonitrile and centrifuged at 300× *g*/3 min/RT, which was followed by two washes with 200 µL of 0.1% TFA in water and centrifuged at 300× *g*/3 min/RT; then, they were left to hydrate for 15 min at RT and centrifuged at 300× *g*/3 min/RT. Peptide solutions were pipetted onto the columns and centrifuged at 500× *g*/3 min/RT. Then, the columns were washed three times with 200 µL of 0.1% TFA in water and centrifuged at 500× *g*/3 min/RT. The elution was performed by the addition of 200 µL of 0.1% TFA in 50% acetonitrile and centrifugation at 500× *g*/3 min/RT, which was followed by 200 µL of 0.1% TFA in 80% acetonitrile and centrifugation under the same conditions, and addition of 200 µL of 0.1% TFA in 100% acetonitrile and centrifugation at 500× *g*/3 min/RT. Eluates were pooled and dried under vacuum. Four pooled samples were prepared for spectral library generation, each pooled from aliquots of tumor or normal samples within the R and NR groups.

#### 2.2.4. Liquid Chromatography (LC)-MS Analysis of Peptides

LC-MS/MS analysis of peptide samples was performed using the RSLCnano system (Thermo Fisher Scientific, Waltham, MA, USA) online connected to an Impact II Ultra-High Resolution Qq-Time-Of-Flight (Bruker, Bremen, Germany) mass spectrometer. Peptides were preconcentrated online on a 100 μm × 30 mm trapping column packed with 3.5-μm X-Bridge BEH 130 C18 (Waters, Milford, MA, USA) prior to LC separation. Equilibration of the trapping and analytical column was performed before the sample injection. Peptides were separated using an Acclaim Pepmap100 C18 column (3 μm particles, 75 μm × 500 mm, Thermo Fisher Scientific) using the following LC gradient (mobile phase A: 0.1% FA (formic acid) in water, mobile phase B: 0.1% FA in 80% acetonitrile: 300 nL/min; 40 °C). The elution gradient started at 1% of mobile phase B, which increased to 56% over 120 min nonlinearly (40 min: 14%, 80 min: 30%, 120 min: 56%) followed by the system wash phase. Analytical column outlet was connected to the CaptiveSpray nanoBooster ion source (Bruker, Bremen, Germany). NanoBooster was filled with acetonitrile. MS and MS/MS spectra were measured either in data-dependent mode (DDA) in 4 samples for spectral library generation in 3 injections each or in data-independent mode (DIA) in all 32 tissue samples in single injections for protein and peptide quantification. DDA data were acquired with 3 s long cycle. The mass range was set to 150–2200 *m*/*z* with precursors selection from 300 to 2000 *m*/*z*. Measurement frequency of MS and MS/MS scans were 2 Hz and 4–16 Hz (depending on the precursor intensity). DIA data were measured in *m*/*z* range of 300–1250 with scan time set to 250 ms or 50 ms for the survey or 64 MS/MS scans (variable windows covering *m*/*z* range of 400–1250; see Table S1 for details), respectively.

#### 2.2.5. MS Data Analysis

First, the spectral library was constructed from DDA data searched in MaxQuant version 1.5.8.3. (www.maxquant.org, accessed on 9 June 2017) against the human UniProt/SwissProt proteome database (UniProt version 2018_03 with 20316 sequences downloaded on 25 April 2018) complemented by the iRT protein database (Biognosys, Schlieren, Switzerland) and an internal database of common protein contaminants in Andromeda search algorithm; the default setting for Bruker qTOF was applied. The enzyme name: Trypsin (cleaving polypeptides at the carboxyl side of lysine or arginine), max. missed cleavage sites 2, taxonomy: *Homo sapiens*. Decoy database search: PSM false discovery rate (FDR) 0.01, protein FDR 0.01. Tolerances: 0.07 Da/0.006 Da (first search/main search) peptide tolerance and 40 ppm IT MS/MS fragment match tolerance. Modifications: Dynamic (variable): oxidation (M), acetyl (protein N-term). Static (fixed): Carbamidomethyl (C). The spectral library was created in Spectronaut 13.9 software (Biognosys) based on a MaxQuant search of all DDA analyses. Quantitative data from DIA runs were obtained in Spectronaut 13.9 for all proteins/peptides/transitions using default settings (FDR = 0.01). The peptides identified with q ˂ 0.01 in at least 8 of 32 analyses were included into the final dataset (q-value percentile 0.25 setting). Differential abundance testing was performed using Student’s *t*-test in Spectronaut 13.9, proteins with absolute log2 fold change (|log2FC|) ˃ 0.58 and with q ˂ 0.05 were considered differentially abundant between sample groups.

#### 2.2.6. Functional Analysis of Identified Proteins

In total, 1996 quantified proteins were functionally analyzed in Gene Set Enrichment Analysis (GSEA) software ver. 4.1.0 (www.gsea-msigdb.org, accessed on 22 October 2020) based on protein group log2FC using the GSEAPreranked tool and searched against hallmarks v7.2., biocarta v7.2., reactome v7.2. and gene ontology (GO) v7.2. databases. Only signaling pathways with FDR q < 0.05 were considered significantly de-regulated.

### 2.3. Cellular Studies

#### 2.3.1. Cell Cultures and Cultivation

786-0, CAKI-1, A704, RCC-MF, RCC-FG2 and RCC-KP cell lines were purchased from CLS Cell Line Services GmbH, Eppelheim, Germany. 786-0, RCC-FG2, and RCC-KP cells were cultivated in RPMI1640 medium, CAKI-1, and A704 in EMEM medium and RCC-MF in high-glucose RPMI1640 medium each supplemented with 10% fetal bovine serum, 1% penicillin–streptomycin (all Sigma-Aldrich, Darmstadt, Germany) in 37 °C in humidified atmosphere of 5% CO_2_.

#### 2.3.2. Immunoblotting

The transgelin level normalized to actin was determined using SDS-PAGE with immunoblotting as described previously [27] with the following modifications: 20 µL of cell lysates prepared in hot (95 °C) complete sample buffer (0.125 M Tris-HCl pH 6.8, 4% SDS, 20% glycerol, 10% mercaptoethanol, 0.005% bromphenol blue) were loaded per well. The detection of transgelin was performed with rabbit polyclonal anti-transgelin antibody (Abcam ab14106; Cambridge, UK; dilution 1:1000), while anti-actin mouse monoclonal antibody clone AC-40 (Sigma-Aldrich A4700; Darmstadt, Germany; dilution 1:1000) was used as loading control. Chemiluminescence was detected by a Vilber Fusion FX7 detection system (Vilber Lourmat Sté, Collégien, France). The semiquantitative densitometry of chemiluminescence bands was performed in Quantity One 4.6 software (Bio-Rad). The transgelin/actin relative optical density (ROD) ratio was calculated as a ratio of integral optical density of transgelin to actin bands. 

#### 2.3.3. CRISPR/Cas9 Gene Editing

Guide RNAs (gRNA) targeting exon2 of the *TAGLN* gene were designed using on-line GeneArt CRISPR Search and Design Tool (https://www.thermofisher.com/cz/en/home/life-science/genome-editing/geneart-crispr/geneart-crispr-search-and-design-tool.html, accessed on 28 November 2018) and synthetized using GeneArt Precision gRNA Synthesis Kit (MAN0014538). Sequences of gRNA were gRNA-1: 5′-ATCGAGAAGAAGTATGACG-3′ and gRNA-2: 5′-CGCGGCTCATGCCATAGGA-3′ in positions 51 nt forward and 37 nt reverse from ATG, respectively. Then, 125 ng of each gRNA were mixed with 500 ng of Cas9 nuclease and transfected into 786-0 cells (starting number of cells 1.2 × 10^4^ per 24-well) using Lipofectamine CRISPRMax according to the manufacturer’s protocol (MAN0014545, Thermo Fisher Scientific). A portion of the cells was lysed 48 h after the transfection to prepare a template for PCR amplification of the *TAGLN* exon2 modified locus. Primers for amplification of the modified locus were: forward primer 5′-CTATGGCTGCTTTCACACTGCA-3′ and reverse primer 5′-GCATAGGACGCAGCTTAGCAGT-3′. DNA modification was analyzed by a DNA hybridization of PCR products and by digesting heteroduplexes with restriction endonuclease using a GeneArt Genomic Cleavage Detection Kit (MAN0009849; Thermo Fisher Scientific) [28]. Heteroduplexes emerged when amplicons with parental and modified sequences were presented in the mixture. Then, the non-complementary sites in heteroduplexes were cleaved by restriction endonuclease, resulting in specific fragments of PCR products. All steps were visualized by agarose electrophoresis. Efficiency of DNA change (%) was calculated as (1 − (1 − Σ of relative intensities of cleaved bands)^1/2^) × 100 [28]. See Figure 1B for CRISPR workflow.

#### 2.3.4. Monoclonal Selection of CRISPR Modified Cells and Their Cultivation

The population of cells with changed DNA was counted. Cells were diluted to seed just one cell per well in a 96-well plate to perform monoclonal selection 72 h after transfection. No selection marker was included in the CRISPR method. Cells were cultivated to grow the colonies and populations. Each colony was tested for transgelin level using SDS-PAGE and immunoblotting as described above. The transgelin/actin ROD ratio was normalized to parental cells, and clones with normalized ROD < 0.5 were considered as clones with reduced transgelin level based on presumption that the silencing of one *TAGLN* gene allele leads to a half decrease in transgelin protein level. The growth time of CRISPR clones was calculated as time from cell seeding until colony harvesting. The DNA of clones with reduced transgelin levels was amplified as described above, and PCR products were sequenced. The clone with modified DNA was further cultivated and analyzed.

#### 2.3.5. siRNA Transfection, Cultivation of siRNA-Transfected Cells, and Scratch Assay

5 × 10^5^ 786-0 cells were transfected with 200 pmol of ON-TARGETplus Human TAGLN siRNA (J-003714-09) or ON-TARGETplus Non-targeting Pool siRNA (D-001810-10; both GE Healthcare-Dharmacon, Lafayette, CO, USA) in transfection buffer (120 mM phosphate buffer pH 7.2, 4 mM KCl, 10 mM MgCl_2_, 10 mM NaCl) using AMAXA Nucleofector IIb (Lonza, Basel, Switzerland). Cells were cultivated 72 h after the transfection; then, they were trypsinized, counted, and 8.4 × 10^4^ cells were seeded per well in technical triplicates in a 24-well plate and cultivated for 24 h. The number of cells grown in a 72 h period was compared between conditions. Scratch assay was performed the next day. Photos of scratches were acquired every two hours until the scratches were covered by cells again by a BEL EureKam CMOS 5.0MPix camera (BEL Engineering, Monza, Italy) connected to a NIB-100 inverted microscope (Novel Optics, Nanjing, China). The photos were analyzed in BEL Capture software version 3.9.0.605 (BEL Engineering). Three independent experiments were performed. 

### 2.4. Statistical Analysis

GraphPad PRISM9 software (version 9.1.0 (221)) was used for all the statistical analyses. Student’s t-test was performed, and *p*-values ˂ 0.05 were considered statistically significant for all experiments. Data from cellular analyses (cell counting, immunoblotting, migration) were reported as the mean ± standard deviation.

## 3. Results

### 3.1. Spectral Library and Mass Spectrometry Data Matrix

To obtain quantitative protein-level data from the DIA-MS dataset, we first generated a spectral library based on triplicate measurements of pooled aliquots of all samples from both tumor and normal tissues from patients responding vs. non-responding to sunitinib treatment in data-dependent acquisition mode. The spectral library was constructed in Spectronaut software based on MaxQuant search results and contained 2357 protein groups represented by 14,294 peptides and their modified variants as well as 15,810 precursors (FDR = 0.01; for a complete list of assays, please see our PRIDE dataset). The library was used for the extraction of quantitative data from the DIA-MS dataset of individually measured tumor tissue samples of eight sunitinib responders (R), eight sunitinib non-responders (NR), and 16 paired adjacent non-tumor (N) tissues. Using this approach, 1996 protein groups, 11,646 peptides and their modified variants, and 12,672 precursors were quantified (q ˂ 0.01) in at least eight analyses that represent one sample group. 

### 3.2. Proteins Associated with Sunitinib Non-Responders

To find proteins associated with each patient’s response to sunitinib treatment, we primarily compared protein levels in tumor tissues from non-responders vs. responders to sunitinib (NR vs. R group), while the group of adjacent non-cancerous tissues (N group) served as a negative control (for the complete lists of proteins in particular comparisons, see Supplementary File S2A–C). Proteins with |log2FC| ˃ 0.58 and q ˂ 0.05 were considered differentially abundant. The 27 most relevant protein groups were differentially abundant both between NR vs. R and in NR vs. N groups and statistically unchanged between R vs. N. Of these, 17 were up-regulated, and 10 were down-regulated in the NR vs. R group (Table 2). The top five most significantly up-regulated proteins associated with poor sunitinib response were lactotransferrin (LTF), transgelin (TAGLN), clusterin (CLU), alpha-1-acid glycoprotein 2 (ORM2), and S100A9, while the top five most significantly down-regulated proteins associated with good sunitinib response were glutathione *S*-transferase A2 (GSTA2), acetyl-coenzyme A synthetase 2-like (ACSS1), protein phosphatase methylesterase 1 (PPME1), dynein light chain 2 (DYNLL2), and heat-shock 70 kDa protein 12A (HSPA12A). The up-regulated proteins associated with poor sunitinib response were mostly localized in extracellular space, while the down-regulated proteins associated with good sunitinib response were mainly intracellular in various organelles (https://www.uniprot.org/, accessed on 15 October 2020). After assessing the roles of these proteins in cancer development and cellular localization, transgelin was selected for further functional characterization in RCC cell lines, as it plays a key role in cytoskeleton remodeling, has been previously associated with various cancer characteristics, and has intracellular localization.

### 3.3. Hallmark and BIOCARTA Pathways, Reactome, and GO Associated with Sunitinib Non-Responders

**Figure 2:**
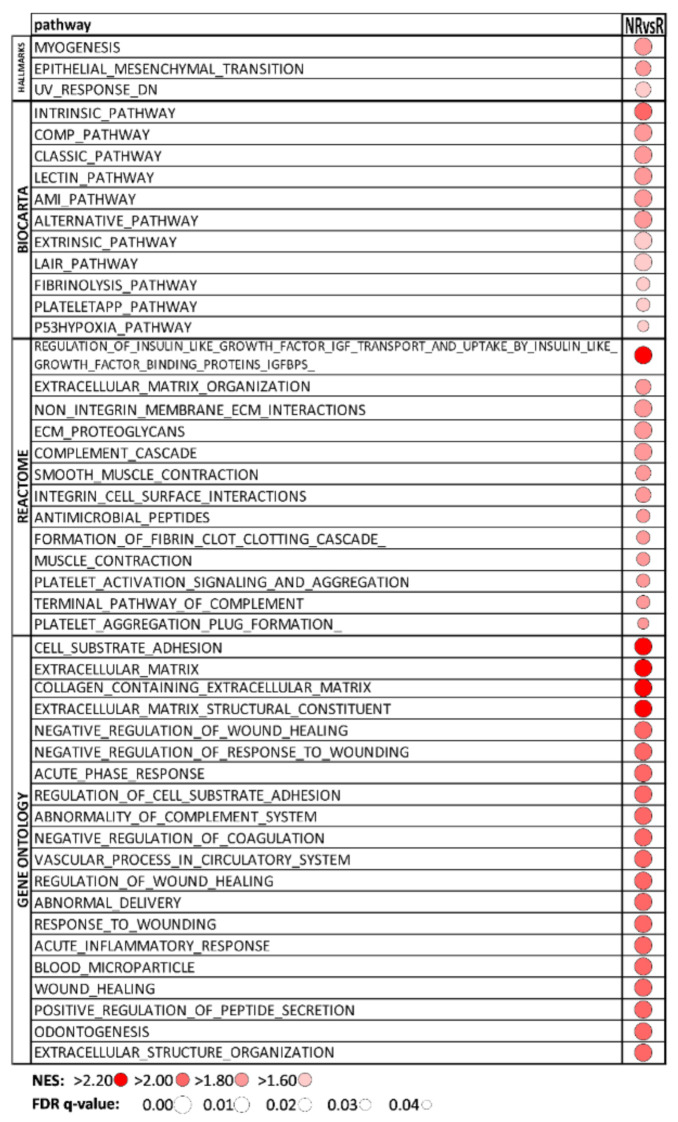
GSEA results: significantly (q < 0.05) positively enriched pathways in NR vs. R comparison. All significant pathways in Hallmark, BIOCARTA and Reactome databases are showed; the top 20 significant pathways are presented in Gene Ontology. NR—non-responders, R—responders, NES—normalized enrichment score.

All 1996 quantified proteins were analyzed for NR vs. R comparison in GSEA software. The list of significantly enriched pathways (q < 0.05) is presented in Figure 2, and interestingly, it contained only positively enriched pathways. A search in the Hallmark database revealed an enrichment of the MYOGENESIS and EPITHELIAL_MESENCHYMAL_TRANSITION pathways. These pathways share nearly one-third of positively enriched proteins together, including transgelin. Positively enriched pathways in BIOCARTA showed extensive changes in immune response and blood clotting/coagulation pathways, supported by results of Reactome and GO databases search. The enrichment was also found in inflammation and hypoxia associated with the p53 protein. In the Reactome and GO search, remodeling of extracellular matrix (ECM), protein interaction in ECM, response to wounding, and up-regulation of the proliferative insulin-like growth factor binding-protein 3 (IGFBP3) pathway were strongly enriched. These changes together significantly support aggressive mccRCC tumor behavior that may modulate response to sunitinib in the NR group

### 3.4. Proteins Differentially Abundant in mccRCC Tumors vs. Adjacent Non-Cancerous Tissue

To find proteins specific for mccRCC tissue, we compared protein levels in both tumor groups (NR + R = T) to those of non-cancerous tissue (N). Similar to NR vs. R comparison, proteins with |log2FC| ˃ 0.58, q ˂ 0.05 were considered statistically significant. In total, we found 789 proteins differentially abundant, of which 323 proteins were up-regulated and 466 proteins were down-regulated in T vs. N tissues (Supplementary File S2D). Most interestingly, we confirmed all seven proteins previously identified in two signatures by Neely et al. and Song et al. as highly expressed in late-stage tumor tissue [29] and associated with the metastasis of mccRCC tumors [30]: perilipin-2, l-lactate dehydrogenase A chain, nicotinamide *N*-methyltransferase, annexin A4, cofilin-1, profilin-1, and fructose-bisphosphate aldolase A (Table S2). These results further confirm the general validity of our data.

### 3.5. Pathways Enriched in mccRCC Tumors Compared to Non-Cancerous Tissues

All 1996 proteins were analyzed in GSEA for T vs. N comparison (Table S3). GSEA revealed pathways cooperating together in RCC tumorigenesis: enrichment of EMT, modifications of immune system signaling through various cytokines, HYPOXIA, which further affects positively ANGIOGENESIS and GLYCOLYSIS and negatively OXIDATIVE PHOSPHORYLATION pathways, TNFα signaling causing enhancement of NF-kB activity and APOPTOSIS and decreasing of ADIPOGENESIS, and concurrent negative enrichment of FATTY ACID and XENOBIOTIC METABOLISMS pathways in the HALLMARK database, all with q < 0.05. The data highly overlap to pathway analysis results of mccRCC tumors by Neely et al. [29] and confirmed metabolic changes on protein level during the tumorigenesis of mccRCC. 

### 3.6. CRISPR/Cas9 Knock-Down Shows That Transgelin Is Essential for the Proliferation of 786-0 RCC Cells 

**Figure 3:**
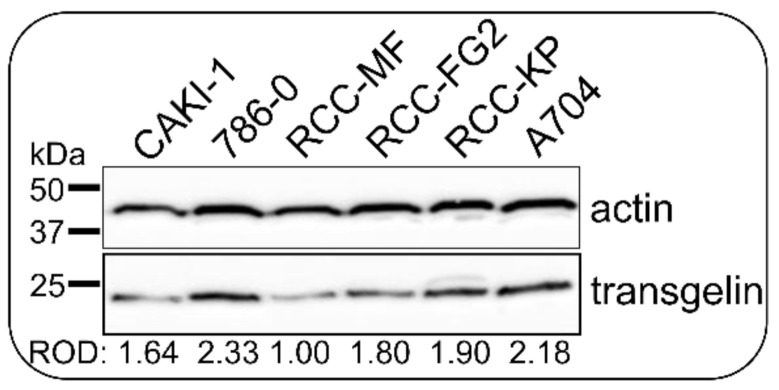
Level of transgelin protein in the panel of ccRCC cell lines. The transgelin level was tested in 20 μg of total protein lysate using immunoblotting. The transgelin/actin ROD ratio was normalized to the RCC-MF cell sample. The highest ROD was detected in 786-0 cells, which were then used in the functional characterization of transgelin.

Transgelin was the second top up-regulated protein, and it was the only one with cytosol localization and a well-known association with tumor biology. Prior to functional characterization, its protein level was tested in a panel of six RCC cell lines that were selected according to the clinicopathological characteristics of original tumors and their cellular properties (Figure 3). The highest transgelin protein level was found in 786-0 cells derived from primary ccRCC carcinoma that were selected for functional characterization. The *TAGLN* gene was disrupted using CRISPR/Cas9 to investigate the impact on cell proliferation and migration. Three independent CRISPR experiments were performed. 786-0 cells were transfected in the first and the second experiment. As we did not obtain any clones with complete *TAGLN* silencing, we used the 786-0 clone C2 carrying one changed *TAGLN* allele after the first CRISPR experiment for the transfection in the third experiment. Cells were transfected with Cas9/anti-transgelin gRNA complexes, and the efficiency of DNA modification was calculated as 31.64%, 29.46%, and 75.45% of total *TAGLN* exon2 DNA sequence based on UV intensities of fragments that have arisen after *TAGLN* exon2 heteroduplexes cleavage in each experiment, respectively (Figure 1C). Monoclonal selection was performed after each CRISPR transfection. Individual survival rates in monoclonal selection were 34.38% (33 of 96 cells), 23.44% (45 of 192 cells), and 38.54% (74 of 192 cells) of seeded cells. In total, 123 clones from all three monoclonal selections were tested for transgelin protein level using semiquantitative densitometry of immunoblots (Figure 1D). Of these 123 clones, 27 clones (21.95%) showed reduced transgelin levels with ROD ˂ 0.5 normalized to parental 786-0 cells, while 96 clones had normal transgelin levels with ROD ≥ 0.5. In general, clones with reduced transgelin levels grew slower (43.67 ± 21.54 days to 100% confluence) in comparison to clones with normal transgelin levels (36.35 ± 9.70 days; *p* = 0.013; Figure 1E). Of the 27 clones with reduced transgelin levels, six clones were sequenced to detect changes in *TAGLN* exon2 DNA, and only one clone C2 was found to have an indel modification in exon2 in one allele of the *TAGLN* gene; the other three clones died in cultivation, when they stopped dividing and cells completely lost their spindle shape toward a larger and flatter one. There was not any clone that kept the reduced transgelin level through the duration of cultivation or which survived with the completely disrupted *TAGLN* gene. As an alternative CRISPR approach, we transfected 786-0 cells with plenticrispr v2 carrying sequences for TAGLN gRNA, Cas9 enzyme, and puromycin resistance [31,32]. After puromycin selection, unfortunately, there was no clone with completely silenced transgelin expression (Supplementary Figure S1). Both CRISPR approaches were also applied in another renal cancer cell line, CAKI-1, with similar results as in 786-0 cells (Supplementary Figure S2A,B). Based on the results above, it is evident that transgelin is an essential protein in ccRCC cells that is necessary for their proliferation, and the cells are unable to grow if transgelin is substantially silenced.

### 3.7. Transient Silencing of Transgelin Slows the Proliferation of 786-0 Cells Down

**Figure 4:**
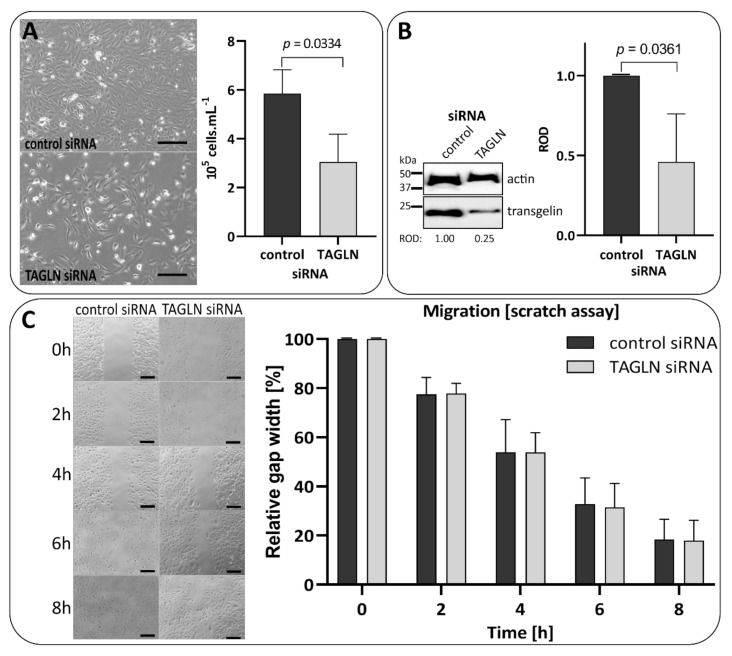
siRNA-based transgelin silencing slows the proliferation of 786-0 cells down. (**A**) 786-0 cells transiently transfected with control siRNA and TAGLN siRNA, 72 h post transfection. The confluence of the TAGLN siRNA-transfected cells was visually lower (**left**), and the difference in cell counts between control siRNA and TAGLN siRNA-transfected cells was significant (**right**). (**B**) Immunoblot of transgelin protein level in siRNA-transfected cells. Transgelin/actin ROD ratio was normalized to control siRNA sample. The difference in ROD ratios between control and TAGLN siRNA-transfected cells was statistically significant. (**C**) Scratch assay was scratched 96 h post transfection (three technical replicates per condition), scratches were monitored every 2 h for 8 h. The scratches were measured in five different positions, values were recalculated to percentage, normalized, and statistically evaluated. The migration rate of TAGLN siRNA-transfected cells was similar as in the control cells, and the difference was insignificant. Representative photos and immunoblots are shown; the graphs are cumulative from three independent biological experiments (for particular results, see Supplementary Figure S3). The black rows in cell photos represent 20 μm.

To independently confirm how partial transgelin silencing affects RCC cell proliferation, we performed three independent transfections of TAGLN siRNA into 786-0 cells. These experiments showed that the proliferation of cells with silenced transgelin (3.051 ± 1.131 × 10^5^ mL^−1^ cells) decreased significantly compared to control siRNA-transfected cells (5.852 ± 0.978 × 10^5^ mL^−1^ cells; *p* = 0.0334; Figure 4A, Supplementary Figure S3A). The transgelin protein level was successfully silenced 72 h post transfection, and its silencing was assessed using semiquantitative densitometry of immunoblots (TAGLN ROD 0.460 ± 0.312 vs. control ROD 1.000 ± 0.000; *p* = 0.0361; Figure 4B, Supplementary Figure S3B). The change in migration between control and TAGLN siRNA-transfected cells was minimal and non-significant, as shown by scratch assays results (Figure 4C, Supplementary Figure S3C). These data further support the previous observation that transgelin is essential for the proliferation of RCC cells. siRNA experiments were also performed in CAKI-1 cells; however, these cells exhibited very low siRNA transfection efficiency and no change in transgelin protein level (Supplementary Figure S4).

## 4. Discussion

### 4.1. Transgelin Is the Key Identified Protein in Intrinsic Sunitinib Resistance through Proliferation Support and EMT

In our study, the transgelin level was found increased in mccRCC tumors non-responding to sunitinib compared to responders, which suggested possible transgelin direct or indirect involvement in intrinsic sunitinib resistance. The functional data based on CRISPR and RNAi clearly confirmed that transgelin is essential for the proliferation of RCC cells, and it is also an important parameter of tumor cell aggressiveness and their ability to form metastases. Transgelin, a 22 kDa protein from the calponin family, plays an active role in actin remodeling and cross-linking, especially in fibroblasts and in smooth muscle cells [33]. Its expression in mature kidney is normally observed only in the vascular smooth muscle cells of vessel walls [34,35]. The increasing expression of transgelin was repeatedly detected in podocytes, parietal, and tubular cells during initiation of nephritis and various types of nephropathies [34,36,37] and in the mesenchymal cells of renal tumor stroma [35]. Higher transgelin levels were associated with the progression of advanced or high-grade disease in colorectal [38,39], breast [40], bladder [41], pancreatic [42], and lung [43] tumors. Transgelin up-regulation in lung tumors was connected with chemoresistance and worse overall survival [44]. A loss of transgelin led to the disruption of actin cytoskeleton organization and aberrant actin filament distribution [38]; this may result in mitotic division blockade. A decreased proliferation rate was also observed in other cell lines with silenced transgelin expression: in SW620 [45], HT-29 and RKO [38] colorectal, MDA MB 231 breast [46], BxPC3 and SW1990 pancreatic [42], and A549 and H358 lung [43] cancer cell lines after silencing transgelin expression by RNAi. Transgelin involvement in myogenesis may be associated with RCC vasculogenesis: transgelin is normally expressed in smooth muscle myocytes surrounding newly emerging vessels [34,35]. As a TGFβ downstream gene, transgelin is implicated in EMT [47] and is often de-regulated in various types of tumors [33]. This was well supported in our GSEA results, where transgelin was the third top up-regulated protein in the EMT pathway. Transgelin in complex or in interaction with other proteins significantly contributes to EMT initiation and modulation by affecting cell migration and promotion in different tumor types [41,48,49]. Together with proliferation support, its role in EMT may be a key factor that contributes to transgelin’s role in intrinsic RCC resistance.

### 4.2. Other Relevant Proteins in Sunitinib Non-Responding Tumors

Apart from transgelin, 16 other proteins were identified as up-regulated in sunitinib non-responsive mccRCC. Of these, 10 proteins were previously reported to have associations with renal tumorigenesis: an elevated level of lactotransferrin (LTF) was reported in non-responsive mccRCC tumors; however, LTF was observed to block metastasis in ccRCC in other studies [50,51]. Clusterin was elevated in acquired resistance to sorafenib, another rTKI drug, in a mouse model [52] and it promoted the growth and invasion of RCC cells in vitro [53]. Alpha-1-acid glycoprotein 2 (ORM2) is a serum protein that binds synthetic drugs and influences their distribution and availability [54]. Nearly all (99%) of sunitinib up-taken into blood is bound to albumin and ORM2 [55], and only 1% of free drug is available to bind target receptors according to “free drug theory”. We speculate that an increased number of ORM2 molecules can bind more sunitinib molecules, which could decrease the availability of free sunitinib in kidney. A high level of S100A9 protein was associated with an unfavorable prognosis in ccRCC [56]. S100A9 supports proliferation and metastasis in other tumor types; S100A9, hemopexin, and inter-alpha-trypsin inhibitor heavy chain H4 (ITIH4) were identified in saliva of patients with RCC and were suggested as potential biomarkers for disease screening [57]. Plasminogen is a part of a 14-gene redox-related prognostic signature of RCC, and its higher mRNA expression was associated with favorable ccRCC prognosis [58,59]. Similarly, ITIH4 is part of the same 14-genes prognostic signature, while its lower expression is favorable [59]. α-1-antichymotrypsin (SERPINA3) and ceruloplasmin are markers of high-grade RCC disease associated with aggressive tumors with poor outcome [60]. SERPINA3 is one of the ccB phenotype genes in ClearCode34, which is specific for more aggressive and metastatic ccRCC tumor subtypes [61]. The ceruloplasmin level grows continually during ccRCC tumorigenesis [62]. Kininogen 1 secretion into urine was found to be lower in patients with RCC tumor in comparison to the control group [63]. These proteins represent other players that may, directly or indirectly, contribute to mechanisms of intrinsic mccRCC resistance to sunitinib.

### 4.3. Role of Enriched Signaling Pathways in Sunitinib Resistance

Sunitinib non-responsive tumors exhibit increased activity of EMT, modulation of immune system response and blood coagulation pathways, and ECM re-organization. The EMT process was shown to be one of the key steps in ccRCC progression [64,65]. EMT itself promotes tumorigenesis and invasion, and it also influences the response to targeted therapies in RCC [65] and is suspected of sunitinib resistance [16]. Abundant proteins localized mainly in the ECM, which are known EMT activators (e.g., collagen 1 [66], fibulin 5 [67]), or promoters (transgelin [68]) in different human cancers, were observed. Collagens also play roles in ECM organization and re-modeling, which is another substantial step in EMT [69]. Yang et al. [70] analyzed transcriptomic data from mccRCC tumors in comparison to non-metastatic ones. They observed an enhanced activity of complement and coagulation pathways in mccRCC tumors and suggested that these pathways can be related to the tumor metastatic potential. Beuselinck et al. [71] designed a 35-gene signature for molecular classification of mccRCC tumors into four different subgroups, where subgroups ccrcc1 and 4 contained sunitinib non-responders and showed poorer progression-free and overall survivals. Both ccrcc1/4 subgroups mirror the ccB subgroup in a ClearCode-34 study by Brooks et al. [61]. Ccrcc4 subgroup pathway analysis was enriched in immune response, chemotaxis, and apoptosis. In our study, immune response (complement system) and coagulation pathways were strengthened in sunitinib-resistant tumors, suggesting the more aggressive behavior of non-responsive tumors and their potential for treatment via immune checkpoint inhibitors. The enrichment of these pathways indicates more aggressive mccRCC tumor behavior that clearly contributes to sunitinib non-responsiveness.

Some studies analyzed individual signaling pathways (mainly angiogenesis [72,73,74,75] or Hedgehog [76]) in association to sunitinib resistance in mccRCC using different methodic approaches, but we did not identify their candidate proteins (e.g., NRP1/2, VEGFR2, RANK/OPG ratio, PD-L1, GLI2) in our proteomic dataset. An elevated protein level of carbonic anhydrase 9 (CA9) in the VEGF pathway was found to be a marker of sunitinib response in mccRCC study [77]. In our study, the trend of CA9 protein level deregulation was the same but statistically insignificant (log2FC = −0.735; q = 0.148). 

The limited number of mccRCC tumors tissues non-responsive to sunitinib and their adjacent normal tissues available in the MMCI Tissue bank is a limitation of our study. The results of our pilot study should be further verified by future studies to overcome possible bias caused by the limited number of available samples. In addition, the massive impact of transgelin CRISPR silencing on RCC cell growth prevented our plan to perform an in vivo study to assess the transgelin role in sunitinib resistance in RCC. However, this study represents the first application of DIA-MS to mccRCC tumors.

## 5. Conclusions

In conclusion, we identified a panel of differentially abundant proteins associated with intrinsic sunitinib resistance in non-responding mccRCC tumors using DIA-MS. In general, up-regulated proteins support proliferation, migration, and invasion, play a role in nutrient supply, and contribute to the aggressive and metastatic behavior of mccRCC tumors. EMT and the other pathways identified as enriched also contributed to the aggressive behavior of sunitinib non-responsive tumors. Intrinsic sunitinib resistance is probably caused by a combination of these factors rather than the action of some proteins. Transgelin is necessary for the proliferation of ccRCC cells, which contributes to aggressive tumor behavior and may be potentially used as a marker of sunitinib non-responsiveness in mccRCC tumors.

## Figures and Tables

**Table 1:** Clinicopathological characteristics of patient cohort.

Categories/Groups		Patients (*n* = 16)	Responders (R, *n* = 8)	Non-Responders (NR, *n* = 8)
sex	M	10	5	5
	F	6	3	3
age at diagnosis, mean (yrs)		63.7 ± 9.5	69.0 ± 5.5	58.4 ± 10.3
Fuhrman grade	1	1	1	0
	2	3	2	1
	3	8	4	4
	4	4	1	3
pT	1a	1	1	0
	1b	3	3	0
	2a	1	1	0
	2b	1	0	1
	3a	5	2	3
	3b	1	0	1
	4	4	1	3
pN	0	12	7	5
	1	2	0	2
	2	2	1	1
mestastases at diagnosis	0	4	4	0
	1	12	4	8
relapse after surgery	yes	4	4	0
	no	12	4	8
sunitinib response	CR	1	1	0
	PR	7	7	0
	PD	8	0	8

R—responders, NR—non-responders, M—male, F—female, CR—complete remission, PR—partial remission, PD—progression disease.

**Table 2:** Proteins specifically deregulated in sunitinib non-responsive vs. responsive mccRCC tumors.

				P vs. R		P vs. N		R vs. N	
	Genes	Protein	Uniprot ID	AVG Log2 Ratio	q Value	AVG Log2 Ratio	q Value	AVG Log2 Ratio	q Value
1	LTF	Lactotransferrin	P02788	2.445	0.004	2.237	0.001	−0.208	0.531
2	TAGLN	Transgelin	Q01995	2.407	0.007	2.667	0.021	0.260	0.328
3	CLU	Clusterin	P10909	2.235	0.029	2.781	0.041	0.546	0.242
4	ORM2	Alpha-1-acid glycoprotein 2	P19652	1.623	0.020	2.850	0.008	1.227	0.516
5	S100A9	Protein S100-A9	P06702	1.553	0.035	2.659	0.006	1.106	0.397
6	CFH	Complement factor H	P08603	1.542	0.019	2.936	0.000	1.395	0.228
7	SERPINA3	Alpha-1-antichymotrypsin	P01011	1.491	0.002	2.906	0.000	1.415	0.162
8	HPX	Hemopexin	P02790	1.400	0.000	2.616	0.000	1.216	0.055
9	PLG	Plasminogen	P00747	1.345	0.001	3.213	0.000	1.868	0.201
10	SERPINC1	Antithrombin-III	P01008	1.309	0.024	1.831	0.015	0.521	0.517
11	KNG1	Kininogen-1	P01042	1.180	0.007	2.403	0.000	1.223	0.111
12	SERPINA1	Alpha-1-antitrypsin	P01009	1.150	0.000	2.420	0.000	1.270	0.475
13	MYH11	Myosin-11	P35749	1.133	0.000	2.197	0.000	1.064	0.130
14	CP	Ceruloplasmin	P00450	1.108	0.003	2.660	0.000	1.553	0.060
15	ITIH4	Inter-alpha-trypsin inhibitor heavy chain H4	Q14624	0.888	0.029	1.865	0.011	0.977	0.396
16	IGHM	Immunoglobulin heavy constant mu	P01871	0.826	0.025	2.562	0.000	1.735	0.181
17	CLTC	Clathrin heavy chain 1	Q00610	0.784	0.001	1.075	0.007	0.291	0.373
18	ERP29	Endoplasmic reticulum resident protein 29	P30040	−0.610	0.039	−1.019	0.004	−0.409	0.209
19	NDUFB11	NADH-ubiquinone oxidoreductase ESSS subunit	Q9NX14	−0.617	0.046	−1.765	0.010	−1.148	0.129
20	HSD17B4	Peroxisomal multifunctional enzyme type 2	P51659	−0.618	0.003	−0.841	0.000	−0.223	0.148
21	DNPH1	2′-deoxynucleoside 5′-phosphate *N*-hydrolase 1	O43598	−0.785	0.036	−0.751	0.025	0.034	0.355
22	CTSC	Dipeptidyl peptidase 1	P53634	−0.804	0.019	−0.969	0.001	−0.165	0.406
23	HSPA12A	Heat shock 70 kDa protein 12A	O43301	−0.806	0.025	−0.856	0.002	−0.050	0.408
24	DYNLL2	Dynein light chain 2, cytoplasmic	Q96FJ2	−0.858	0.039	−1.066	0.001	−0.208	0.222
25	PPME1	Protein phosphatase methylesterase 1	Q9Y570	−0.889	0.022	−3.817	0.048	−2.928	0.260
26	ACSS1	Acetyl-coenzyme A synthetase 2-like, mitochondrial	Q9NUB1	−1.666	0.041	−1.816	0.021	−0.150	0.503
27	GSTA2	Glutathione *S*-transferase A2	P09210	−2.219	0.000	−1.776	0.000	0.443	0.389

NR—non-responders, R—responders, N—normal.

## Data Availability

The mass spectrometry proteomics data and spectral library have been deposited in the ProteomeXchange Consortium via the Proteomics Identifications (PRIDE) partner repository (http://www.ebi.ac.uk/pride/archive/, accessed on 26 August 2021) with the dataset identifier PXD027065.

## Supplementary Materials

The following are available online at https://www.mdpi.com/article/10.3390/biomedicines9091145/s1, File S1: Detailed information on patients involved in the study; File S2: List of quantified proteins in NR vs. R (A), NR vs. N (B) and R vs. N (C) tissues and in T vs. N tissue comparison (D); Table S1: Transition list in DIA-MS measurement; Table S2: Seven key proteins differentially abundant in T vs. N comparison identified also in other RCC analyses using MS; Table S3: GSEA results: significantly (q < 0.05) positively and negatively enriched pathways in T vs. N comparison; Figure S1: Detection of transgelin protein level in clones of the 786-0 cell line after puromycin selection of the 786-0 population transfected with plasmid plenticrispr v2 TAGLN; Figure S2: Detection of transgelin protein level in CAKI-1 cells after CRISPR transfection. (A) The clones after TAGLN gRNA and Cas9 enzyme complex transfection. (B) The clones after puromycin selection of population transfected with plasmid plenticrispr v2 TAGLN. Transgelin protein level did not decrease in any clone. MS, monoclonal selection in a 96-well plate; col, colonies growing in situ on 10 cm cultivation dish; Figure S3: Particular results of three replicates of siRNA transfected 786-0 cells. (A) Cell counts of 786-0 cells 72 h after transfection of control or TAGLN siRNA. (B) Immunoblotting analysis of transgelin 72 h after transfection and ROD semiquantitative analysis of immunoblots. (C) Results of scratch assays; Figure S4: Detection of transgelin protein level in CAKI-1 cells transfected with 200 pmol of anti-TAGLN siRNA and non-targeted control (NT ctrl) siRNA in three independent experiments.
