# Supplementary material for: Transgelin Contributes to a Poor Response of Metastatic Renal Cell Carcinoma to Sunitinib Treatment
Source: Biomedicines. 2021 Sep 3;9(9):1145. doi: 10.3390/biomedicines9091145 (PMC8467952; doi:10.3390/biomedicines9091145)
Supplement: Supplementary file 1 [file biomedicines-09-01145-s001.zip › Supplementary_materials.pdf]

Supplementary table S1. Transition list in DIA-MS measurement.

Scan number	Mass	Width	isCID	Collision	xAcq.
1	400.00	0.0	0	0.0	5
2	403.85	8.7	0	28.6	1
3	411.50	8.6	0	28.9	1
4	418.75	7.9	0	29.2	1
5	425.70	8.0	0	29.5	1
6	433.00	8.6	0	29.7	1
7	440.55	8.5	0	30.0	1
8	447.75	7.9	0	30.3	1
9	454.70	8.0	0	30.5	1
10	462.20	9.0	0	30.8	1
11	469.55	7.7	0	31.0	1
12	475.35	5.9	0	31.2	1
13	481.35	8.1	0	31.4	1
14	487.80	6.8	0	31.6	1
15	493.95	7.5	0	31.8	1
16	500.50	7.6	0	32.0	1
17	507.25	7.9	0	32.2	1
18	514.20	8.0	0	32.5	1
19	521.25	8.1	0	32.7	1
20	528.80	9.0	0	32.9	1
21	536.30	8.0	0	33.1	1
22	543.05	7.5	0	33.3	1
23	550.15	8.7	0	33.5	1
24	557.65	8.3	0	33.7	1
25	564.55	7.5	0	33.9	1
26	571.50	8.4	0	34.1	1
27	578.50	7.6	0	34.3	1
28	585.05	7.5	0	34.5	1
29	591.80	8.0	0	34.7	1
30	598.30	7.0	0	34.9	1
31	604.85	8.1	0	35.0	1
32	612.40	9.0	0	35.2	1
33	620.35	8.9	0	35.4	1
34	628.00	8.4	0	35.6	1
35	636.00	9.6	0	35.8	1
36	643.85	8.1	0	36.0	1
37	650.85	7.9	0	36.2	1
38	657.65	7.7	0	36.4	1
39	665.40	9.8	0	36.5	1
40	674.05	9.5	0	36.8	1
41	682.80	10.0	0	37.0	1

42	691.85	10.1	0	37.2	1
43	701.40	11.0	0	37.4	1
44	710.90	10.0	0	37.6	1
45	720.55	11.3	0	37.8	1
46	731.30	12.2	0	38.0	1
47	741.50	10.2	0	38.3	1
48	751.55	11.9	0	38.5	1
49	762.20	11.4	0	38.7	1
50	773.20	12.6	0	38.9	1
51	785.70	14.4	0	39.2	1
52	799.45	15.1	0	39.5	1
53	813.00	14.0	0	39.7	1
54	826.60	15.2	0	40.0	1
55	841.30	16.2	0	40.3	1
56	857.20	17.6	0	40.6	1
57	874.70	19.4	0	40.9	1
58	891.65	16.5	0	41.2	1
59	908.95	20.1	0	41.5	1
60	930.05	24.1	0	41.9	1
61	956.35	30.5	0	42.3	1
62	988.30	35.4	0	42.8	1
63	1029.00	48.0	0	43.5	1
64	1081.30	58.6	0	44.3	1
65	1155.05	90.9	0	45.3	1

Supplementary table S2. Seven key proteins differentially abundant in T vs. N comparison identified also in other RCC analyses using MS.

	Uniprot IDs	AVG Log2 Ratio	Pvalue	Qvalue	# of Ratios	Genes	Protein	# Unique Total Peptides	% Change	Ratio
1	Q99541	1.175	0.000	0.000	144	PLIN2	Perilipin-2	11	125.850	2.258
2	P00338	1.408	0.000	0.000	288	LDHA	L-lactate dehydrogenase A chain	14	165.418	2.654
3	P09525	2.556	0.000	0.000	384	ANXA4	Annexin A4	18	487.897	5.879
4	P40261	2.470	0.000	0.000	144	NNMT	Nicotinamide N-methyltransferase	11	454.096	5.541
5	P23528	0.717	0.000	0.000	176	CFL1	Cofilin-1	11	64.363	1.644
6	P07737	0.825	0.000	0.000	144	PFN1	Profilin-1	8	77.118	1.771
7	P04075	2.110	0.000	0.000	368	ALDOA	Fructose-bisphosphate aldolase A	18	331.569	4.316

Song et al. [28] and Neely et al. [29] together identified seven proteins differentially abundant in mccRCC tissues, that were also significantly deregulated in our analysis.

Supplementary table S3. GSEA results: significantly (q < 0.05) positively and negatively enriched pathways in T vs. N comparison.

	pathway	TvsR
HALLMARKS	EPITHELIAL_MESENCHYMAL_TRANSITION	
	INTERFERON_GAMMA_RESPONSE	
	INTERFERON_ALPHA_RESPONSE	
	ALLOGRAFT_REJECTION	
	UV_RESPONSE_DN	
	HYPOXIA	
	TNFA_SIGNALING_VIA_NFKB	
	APOPTOSIS	
	IL2_STAT5_SIGNALING	
	ANGIOGENESIS	
	GLYCOLYSIS	
	OXIDATIVE_PHOSPHORYLATION	
	FATTY_ACID_METABOLISM	
	ADIPOGENESIS	
	XENOBIOTIC_METABOLISM	
BIOCARTA	AMI_PATHWAY	
	FIBRINOLYSIS_PATHWAY	
	EXTRINSIC_PATHWAY	
REACTOME	EXTRACELLULAR_MATRIX_ORGANIZATION	
	ECM_PROTEOGLYCANS	
	INTEGRIN_CELL_SURFACE_INTERACTIONS	
	INTERFERON_SIGNALING	
	REGULATION_OF_INSULIN_LIKE_GROWTH_FACTOR_IGF_TRANSPORT_AND_UPTAKE_BY_INSULIN_LIKE_GROWTH_FACTOR_BINDING_PROTEINS_IGFBPS_	
	INTERFERON_ALPHA_BETA_SIGNALING	
	COLLAGEN_BIOSYNTHESIS_AND_MODIFYING_ENZYMES	
	COLLAGEN_FORMATION	
	SIGNALING_BY_PDGF	
	COLLAGEN_DEGRADATION	
	THE_CITRIC_ACID_TCA_CYCLE_AND_RESPIRATORY_ELECTRON_TRANSPORT	
	RESPIRATORY_ELECTRON_TRANSPORT_ATP_SYNTHESIS_BY_CHEMIOSMOTIC_COUPLING_AND_HEAT_PRODUCTION_BY_UNCOUPLING_PROTEINS_	
	RESPIRATORY_ELECTRON_TRANSPORT	
	PROTEIN_LOCALIZATION	
	COMPLEX_I_BIOGENESIS	
	FATTY_ACID_METABOLISM	
	GLYOXYLATE_METABOLISM_AND_GLYCINE_DEGRADATION	
	BRANCHED_CHAIN_AMINO_ACID_CATABOLISM	
	CITRIC_ACID_CYCLE_TCA_CYCLE_	
	PEROXISOMAL_PROTEIN_IMPORT	
GENE ONTOLOGY	EXTRACELLULAR_STRUCTURE_ORGANIZATION	
	EXTRACELLULAR_MATRIX_STRUCTURAL_CONSTITUENT	
	COLLAGEN_CONTAINING_EXTRACELLULAR_MATRIX	
	BLOOD_MICROPARTICLE	
	EXTRACELLULAR_MATRIX	
	ENDOPLASMIC_RETICULUM_LUMEN	
	RESPONSE_TO_TYPE_I_INTERFERON	
	INTERFERON_GAMMA_MEDIATED_SIGNALING_PATHWAY	
	PLATELET_ALPHA_GRANULE_LUMEN	
	CYTOKINE_MEDIATED_SIGNALING_PATHWAY	
	MITOCHONDRIAL_MATRIX	
	ORGANIC_ACID_CATABOLIC_PROCESS	
	CELLULAR_RESPIRATION	
	MITOCHONDRION	
	ABNORMAL_URINE_PH	
	RESPIRATORY_ELECTRON_TRANSPORT_CHAIN	
	ABNORMALITY_OF_ACID_BASE_HOMEOSTASIS	
	ATP_SYNTHESIS_COUPLED_ELECTRON_TRANSPORT	
	CELLULAR_AMINO_ACID_CATABOLIC_PROCESS	
	OXIDOREDUCTASE_COMPLEX	

NES: 2.50 ● 2.20 ● 1.90 ● 1.60 ● -1.60 ● -1.90 ● -2.20 ● -2.50 ●

FDR q-value: 0.00 ○ 0.01 ○ 0.02 ○ 0.03 ○ 0.04 ○

All significant pathways in Hallmark and BIOCARTA databases are showed. Top10 the most positively and negatively enriched pathways in Reactome and Gene Ontology databases are presented. T tumor, N normal, NES normalized enrichment score.

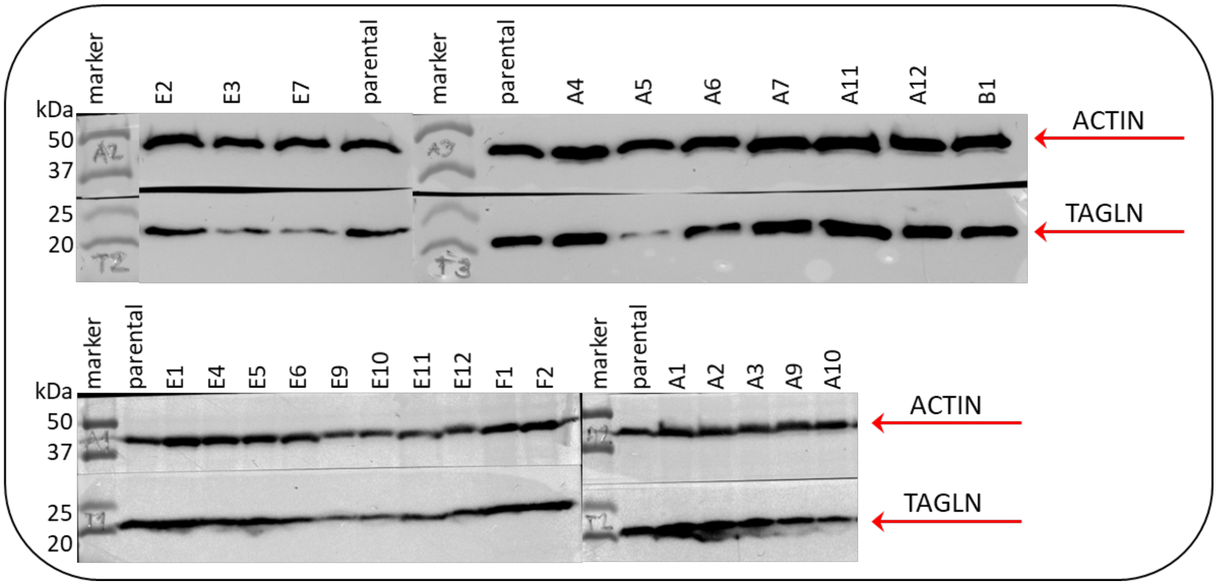

Supplementary figure S1. Detection of transgelin protein level in clones of 786-0 cell line after puromycin selection of 786-0 population transfected with plasmid plenticrispr v2 TAGLN. None of the clones has completely silenced transgelin protein expression and only 3 clones out of 25 exhibit decreased transgelin protein level.

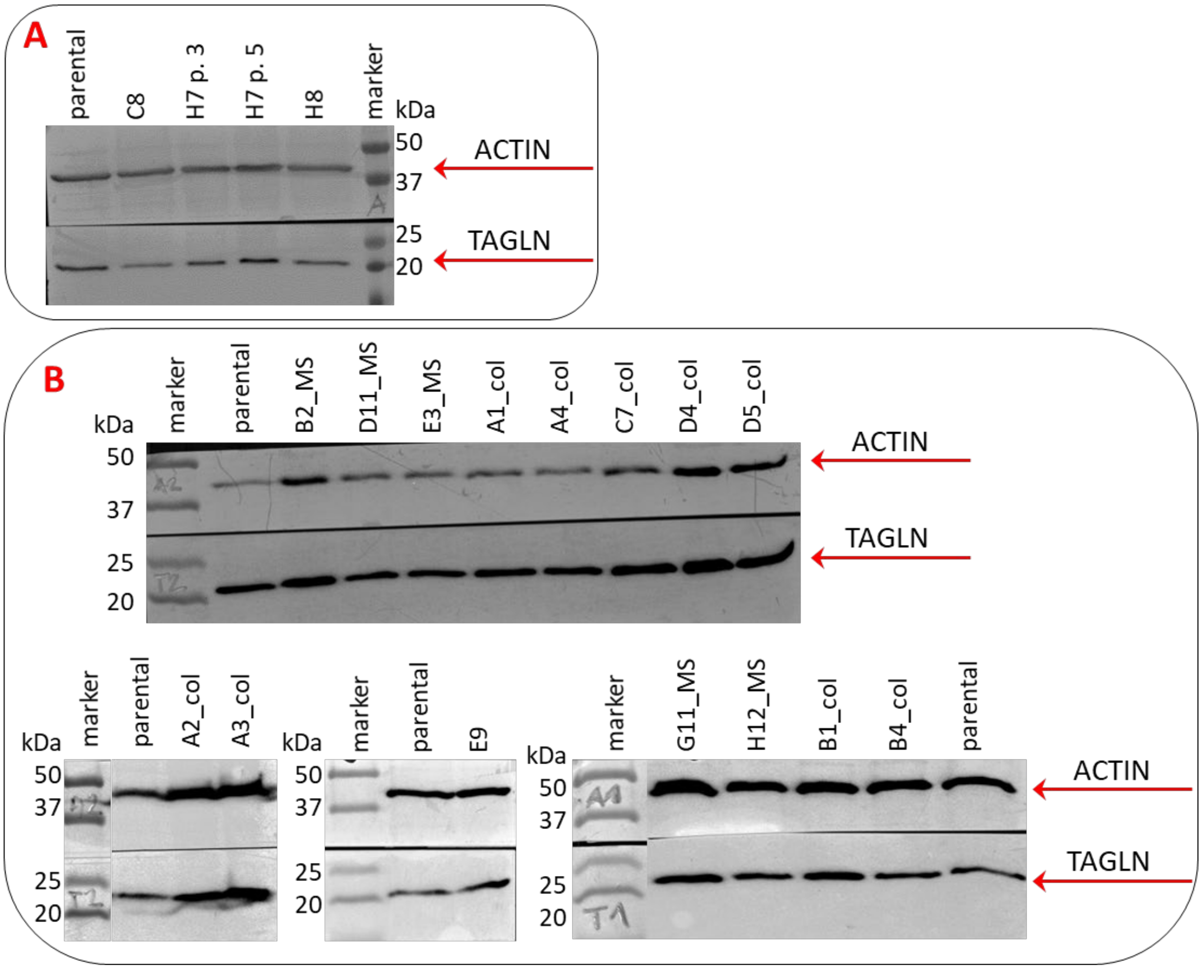

Supplementary figure S2. Detection of transgelin protein level in CAKI-1 cells after CRISPR transfection. (A) The clones after TAGLN gRNA and Cas9 enzyme complex transfection. (B) The clones after puromycin selection of population transfected with plasmid plenticrispr v2 TAGLN. Transgelin protein level did not decrease in any clone. MS, monoclonal selection in a 96-well plate; col, colonies growing *in situ* on 10 cm cultivation dish.

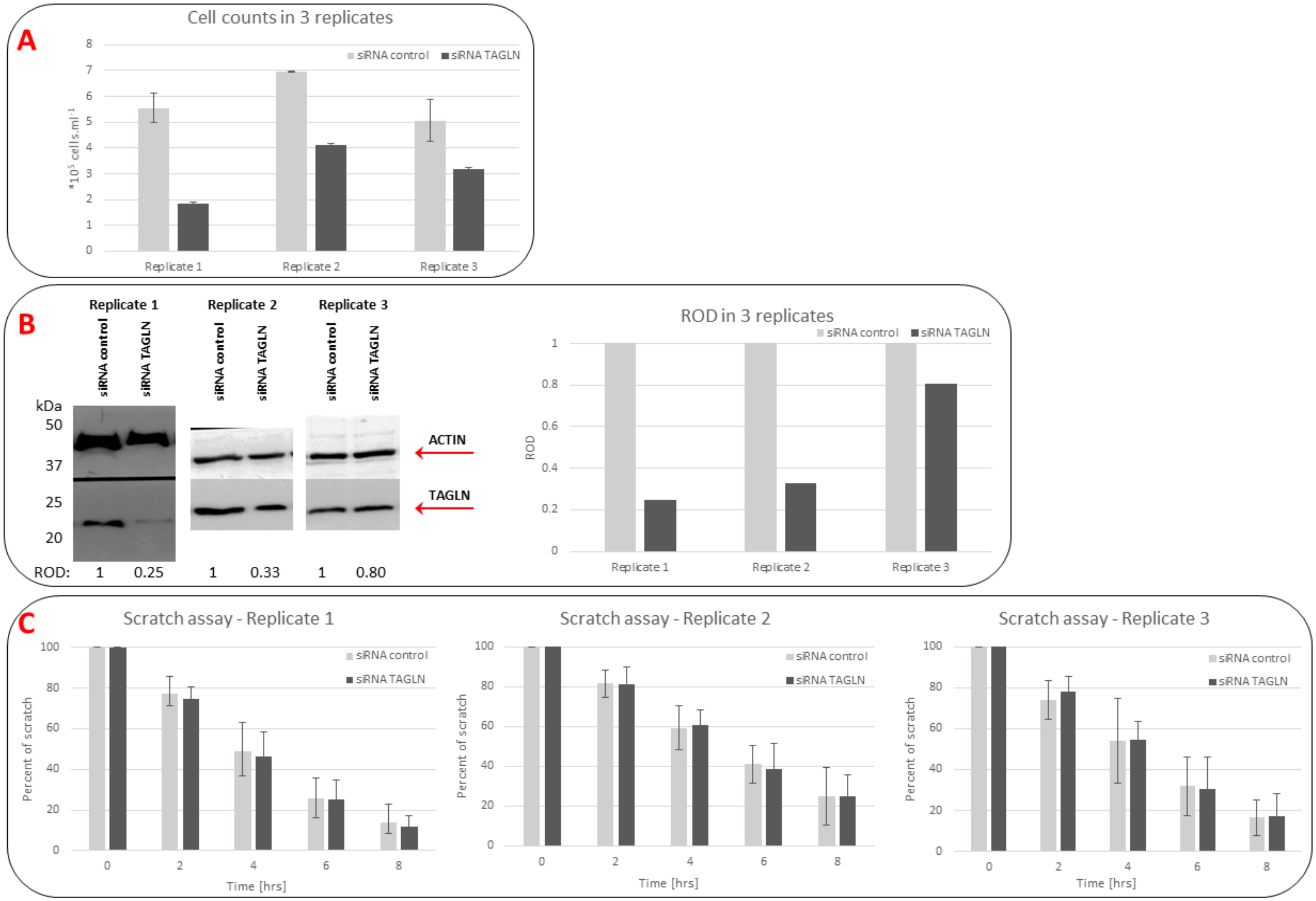

Supplementary figure S3. Particular results of three replicates of siRNA transfected 786-0 cells.

(A) Cell counts of 786-0 cells 72 hours after transfection of control or TAGLN siRNA. (B) Immunoblotting analysis of transgelin 72 hours after transfection and ROD semiquantitative analysis of immunoblots. (C) Results of scratch assays.

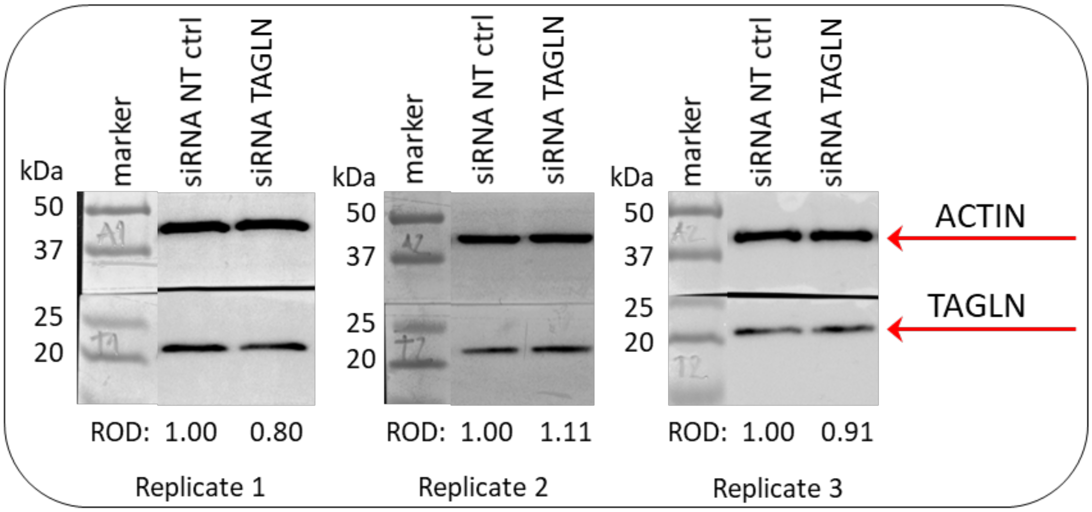

Supplementary figure S4. Detection of transgelin protein level in CAKI-1 cells transfected with 200 pmol of anti-TAGLN siRNA and non-targeted control (NT ctrl) siRNA in three independent experiments. Cells were harvested 48 hours after transfection. ROD, transgelin/actin relative optical density ratio.
